# A Nanotechnology-Ready Computing Scheme based on a Weakly Coupled Oscillator Network

**DOI:** 10.1038/srep44772

**Published:** 2017-03-21

**Authors:** Damir Vodenicarevic, Nicolas Locatelli, Flavio Abreu Araujo, Julie Grollier, Damien Querlioz

**Affiliations:** 1 Centre de Nanosciences et de Nanotechnologies, CNRS, Univ. Paris-Sud, Université Paris-Saclay, C2N – Orsay, Orsay cedex, 91405, France; 2 Unité Mixte de Physique CNRS, Thales, Univ. Paris-Sud, Université Paris-Saclay, Palaiseau, 91767, France

## Abstract

With conventional transistor technologies reaching their limits, alternative computing schemes based on novel technologies are currently gaining considerable interest. Notably, promising computing approaches have proposed to leverage the complex dynamics emerging in networks of coupled oscillators based on nanotechnologies. The physical implementation of such architectures remains a true challenge, however, as most proposed ideas are not robust to nanotechnology devices’ non-idealities. In this work, we propose and investigate the implementation of an oscillator-based architecture, which can be used to carry out pattern recognition tasks, and which is tailored to the specificities of nanotechnologies. This scheme relies on a weak coupling between oscillators, and does not require a fine tuning of the coupling values. After evaluating its reliability under the severe constraints associated to nanotechnologies, we explore the scalability of such an architecture, suggesting its potential to realize pattern recognition tasks using limited resources. We show that it is robust to issues like noise, variability and oscillator non-linearity. Defining network optimization design rules, we show that nano-oscillator networks could be used for efficient cognitive processing.

Synchronization in networks of interacting oscillators is a common phenomenon in our environment. It rises spontaneously, for example, in crowd-clapping, circadian rhythms, business cycles or even firefly colonies^1^. Coupled oscillators synchronization has therefore been studied extensively by mathematicians and theoretical physicists^2^,^3^,^4^,^5^,^6^,^7^,^8^. It was also identified as a likely source of high computational power in the brain, which contains assemblies of interacting oscillating neurons^9^,^10^,^11^,^12^,^13^,^14^,^15^.

Starting in early 1950s, efforts were made to leverage the appealing capabilities of synchronization for computation, with the development of parametrons as logic circuit elements^16^,^17^. These first technologies were eventually abandoned in profit of faster and more scalable Complementary Metal-Oxide Semiconductor (CMOS) technology. Nevertheless, CMOS technology is currently reaching scalability limits^18^. The focus of computing has also shifted toward tasks such as pattern recognition, classification or decision taking, which do not map well on the conventional CMOS-based von Neumann architecture^19^. These two considerations have led to a renewing strong interest for the development of unconventional oscillator-based computing architectures^20^,^21^,^22^,^23^,^24^,^25^. This interest is also fueled by the development of an increasing number of compact and highly integrable oscillator nanotechnologies, which have proven synchronization abilities: superconducting Josephson junctions^26^,^27^, laser oscillators^28^, microelectromechanical systems^29^,^30^,^31^,^32^, spin-torque nano-oscillators^33^,^34^,^35^,^36^,^37^,^38^,^39^, oxide-based oscillators^23^,^40^,^41^. However, natural interactions between such nano-oscillators are usually weak, and have to face issues such as large phase-noise, variability and partly non-linear behaviors^42^,^43^ that can have a negative influence on synchronization^44^,^45^. Their influence must be assessed to design robust oscillator-based computing architectures.

Most architectures proposed for oscillator-based computing perform associative operations. The input is presented as a set of frequencies or initial phases. The network then naturally converges to a synchronization state, which can be taken as output and used for example for recognition or classification. Unfortunately, this general scheme raises challenges for implementation with nanotechnologies. Notably, many architectures require perfect phase synchronization^21^,^24^,^46^,^47^, which is jeopardized by phase noise and variability. Many architectures also require changing the couplings between oscillators during the associative process^21^,^48^, which is very hard to achieve in the context of nanotechnology. An alternative approach proposes to use a global time-dependent signal to emulate a dynamic connectivity in a network of homogeneous interconnections, but this comes at the expense of the generation of a highly complex arbitrary input signal^49^.

In this work, we investigate an oscillator network based computing architecture that avoids these issues and is specially relevant within the constraints of nanotechnologies. It is based on an approach, originally proposed by Vassilieva *et al*. and until now only studied in a computer-based machine learning context^8^, based on a fixed network of weak connections between oscillators, and relying on the control of the natural frequencies of individual oscillators. This architecture avoids the reading and control of individual phases, and can process noisy inputs. Here, we suggest that this architecture can be implemented by leveraging a general property of nano-oscillators allowing convenient frequency tuning through an external bias (usually current or voltage) and that the readout could easily be implemented by pairwise synchronization evaluation using elementary logic circuits. With regards to ref. 8, we investigate the impact of the main issues regarding a nanotechnology implementation: intrinsic noise of the oscillators, variability of their physical properties as well of their coupling strength, presence of non-linearity or phase shifts, impact of the network geometry on the coupling. By the means of comprehensive simulations, we investigate how the architecture tolerates or can be adapted to these issues.

After introducing the architecture and its input and readout protocols, we investigate, through extensive numerical simulations and theoretical analysis, the resilience of such a pattern recognition scheme to phase noise, variability, and non-linearities present in nanotechnologies. We then assess the scaling properties of the network by evaluating the evolution of its pattern discrimination capacity with increasing numbers of oscillators. We complete this study by investigating geometrical effects that can appear in nano-device architectures. Finally, we discuss these results in the realistic context of available nano-devices and in the light of the latest achievements on their synchronization capabilities.

## Results

### System description

**Figure 1:**
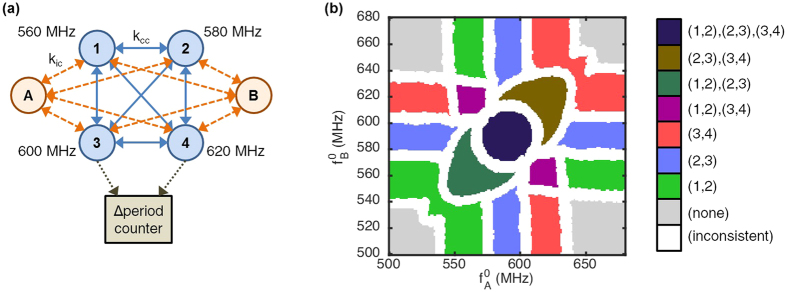
(**a**) Diagram of the oscillator network showing input oscillators A,B and core oscillators 1, 2, 3, 4. (**b**) The output synchronization readout map of the ideal reference oscillator network. Each color represents a different set of synchronized pairs of core oscillators. The gray 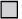 regions represent areas where none of the oscillator pairs is synchronized. The white 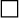 regions correspond to situations where the evaluated synchronization state is sensitive to the initial conditions.

The pattern recognition architecture chosen as the basis of this study is presented in Fig. 1(a). It consists of a core network of oscillators that are coupled by fixed bidirectional weak connections. All-to-all coupling is considered in this study unless stated otherwise. Such all-to-all coupling can be expected in the case electrical coupling of resistive elements^50^ or by summing the signals of all oscillators and re-injecting the total signal by the use of external circuitry^38^. As their natural frequencies are spread, no synchronization between the oscillators happens spontaneously. A set of input oscillators, with stronger couplings to the core oscillators, is used to perturb the core network. The input to the network is encoded as the natural frequency of these input oscillators.

The readout map of Fig. 1(b) illustrates the typical response of a system of 4 core and 2 input oscillators. Under the influence of the inputs (or stimuli), synchronizations between oscillators of the core network emerge. The map shows regions corresponding to the different output synchronization patterns triggered by the choice of input natural frequencies 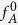 and 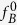. On the sides of the map, only one input oscillator interacts with the core network, as the other one’s natural frequency is very different from the natural frequencies of the core oscillators. This results in the synchronization regions, 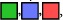, involving the synchronization of a single pair of core oscillators. The central regions, 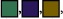, result from the interaction of multiple core oscillators with both input oscillators. They correspond to the synchronization of more than a single pair.

The resulting list of synchronized pairs of core oscillators corresponds to the output synchronization pattern of the system and is strongly dependent on the natural frequencies of the input oscillators. It is therefore a signature of the input stimuli, and can be used to achieve classification/recognition of the presented input.

By performing an associative operation between a set of analog inputs and a limited set of synchronization patterns, this system behaves as a hetero-associative memory. This scheme can be used for different kinds of multi-class classification problems, such as image classification^8^, spoken word classification or decision making. Shaping the response of such a network so that the output can be used for classifying the inputs requires a learning process such the one described in ref. 8.

In this work, except when assessing the impact of oscillator non-linearities, we simulate the oscillator-based architecture by using the Kuramoto equation (1) describing the evolution of oscillator *i*’s phase *θ*_*i*_ as a function of its intrinsic frequency 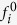 and the influence of the other oscillators 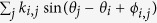. The coupling from oscillator *j* to oscillator *i* is modeled through the coupling strength *k*_*i,j*_ and the coupling phase *ϕ*_*i,j*_. Unless stated otherwise, a phase shift *ϕ*_*i,j*_ = 0 is assumed. When specified, a phase noise term is also included in the simulation.


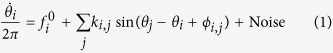


Despite its simplicity, the Kuramoto model captures the advanced dynamics of an oscillator network^3^,^6^,^33^ and is heavily used to model systems as complex as ensembles of interacting neurons^15^, while remaining generic and fit for the description of numerous nanodevices.

We consider that the core network is initially in a random, unsynchronized state before the input stimuli are applied. After applying the stimuli and waiting for the stabilization of the frequencies in the network during a given stabilization time, its state is read by pairwise evaluation of synchronization between core network oscillators. For the sake of reducing complexity, only synchronization between oscillators with consecutive natural frequencies is evaluated, strongly reducing the number of circuits needed for the readout process.

### Reference architecture

To illustrate the robustness of this architecture, we focus on the small scale architecture shown in Fig. 1(a) which uses *N*_*c*_ = 4 core oscillators with natural frequencies 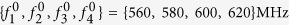 and *N*_*i*_ = 2 input oscillators {*A, B*} with variable frequencies 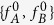 between 500 and 680 MHz. The natural frequencies of oscillators are set either at design time by tuning the material or geometrical properties of each oscillator, and/or by applying a bias assuming the knowledge of the frequency tuning function *f*^0^(bias). A properly scaled input pattern {*v*_1_, *v*_2_} is presented by setting the natural frequencies of the input oscillators 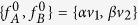. For instance, as illustrated in ref. 8. in the case of image classification, 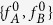 can code for the frequencies of the two main harmonics {*v*_1_, *v*_2_} of an image FFT. The weak core-core coupling constant *k*_*cc*_ = 4 MHz and the stronger input-core coupling constant *k*_*ic*_ = 12 MHz are chosen so that no spontaneous synchronization emerges among the core oscillators in the absence of stimuli, and appropriate response arises when input stimuli are applied. For all simulations, the oscillator network dynamics are computed for 1 *μ*s. The synchronization state is then evaluated during 0.5 *μs* after a 0.5 *μs* stabilization time.

**Figure 2:**
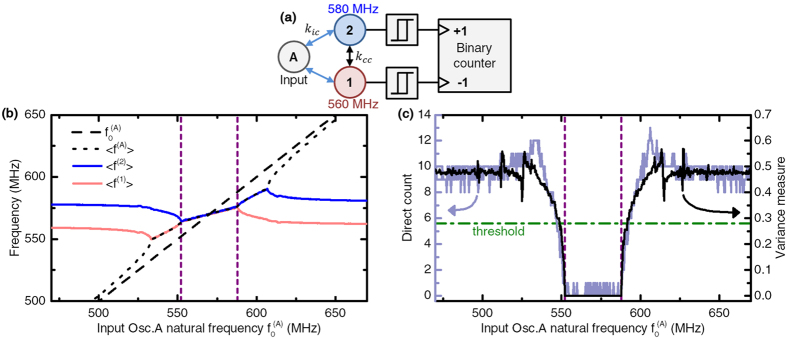
(**a**) Three-oscillator circuit, and direct counter synchronization detector between oscillators 1 and 2. (**b**) Average frequencies of the oscillators in the circuit when the natural frequency of input oscillator A (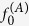) varies. (**c**) Final absolute value of the direct counter as a function of 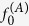, compared to the state of the art variance measure approach. The green horizontal line corresponds to the threshold chosen to discriminate synchronization.

The original proposal of ref. 8. for evaluating synchronization involves the computation of the variance of the sinus of the phase difference between two oscillators. Although this technique is perfectly appropriate for theoretical investigations, this measurement is hard to implement in hardware circuits. Instead, here, we make use of the detector circuit of Fig. 2(a), originally proposed in ref. 51, which uses basic logic counter circuits approximating the average absolute frequency difference between two oscillators and comparing it to a given threshold. The operation of this circuit is illustrated by simulating two oscillators (“1” and “2”) coupled with an input oscillator “A”, and sweeping the frequency of this input oscillator. Figure 2(b) plots the average frequencies 〈* f*^ (A)^〉, 〈* f*^ (1)^〉 and 〈*f *^(2)^〉 of oscillators A, 1 and 2 and shows that oscillators 1 and 2 are synchronized in a narrow band of input frequencies. Figure 2(c) plots the synchronization of the two oscillators, as evaluated by the original variance method and our technique (“Direct count”). The two techniques result in measurements that can almost be superimposed, therefore validating the simple circuit. We also use this graph to define threshold values (green horizontal line) that separate “synchronized” from “unsynchronized” pairs. This definition of synchronization, or quasi-synchronization, does not require perfect phase locking and is particularly relevant in the context of noisy weakly coupled oscillators, and brings robustness to noise.

The complete readout map of the ideal system of Fig. 1(a) was computed on a massively parallel Graphics Computing Unit (GPU, see Methods) for 200 × 200 = 40,000 input natural frequency combinations 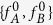, and is shown in Fig. 1(b), where points are colored according to the detected synchronization pairs. For each point, in order to avoid sensitivity to the initial state of the core network, the simulation was repeated 10 times starting with different random initial phases. The point is then blanked if at least one simulation yields a different output synchronization pattern. Finally, a filtering step is applied to the map that blanks out all the points whose pattern does not appear consistently in a 3 MHz-radius region around the point. This step avoids counting spurious readout patterns and guarantees that the obtained synchronization regions are robust to small variations of the presented input frequencies.

As shown in the readout map of Fig. 1(b), the synchronization regions discriminating classes of inputs are fairly wide and clearly defined, which confirms that this approach is appropriate for oscillator-based pattern classification. The sizes and positions of the regions are of course strongly dependent on the choice of natural frequencies of oscillators in the core network as well as their associated frequency differences. This property is exploited as an asset to shape the network classification abilities by adaptation of the oscillator frequencies in ref. 8: the learning operation consists of presenting examples and adjusting the natural frequencies to promote expected synchronizations and break undesired ones. Furthermore, given the natural frequency tuning capabilities of the proposed system, online learning algorithms can also be used. Following this scheme, the proposed architecture only uses natural frequencies as tuning parameters, with fixed couplings, which is highly relevant for the design of nano-architectures.

### Behavior under the presence of noise

Noise is an important challenge in hardware implementations of oscillator-based computing. In the literature, noise was observed to not only prevent synchronization^1^ but also to induce fluctuations in the synchronization pattern readout during evaluation time, showing transitions in regions where multiple synchronization attractors are available^44^. To fully assess the influence of noise in this architecture, we simulate the reference architecture for increasing phase noise levels on both input and core oscillators. We then compare the obtained readout map to the ideal map evaluated in the noiseless case (Fig. 1).

Noise is included in the solved Kuramoto equation 1 and the specific Euler-Maruyama SDE integration scheme is used (see details in Methods section). The noise FWHM is defined to be the Full Width at Half Maximum of the power spectrum density of an isolated oscillator.

**Figure 3:**
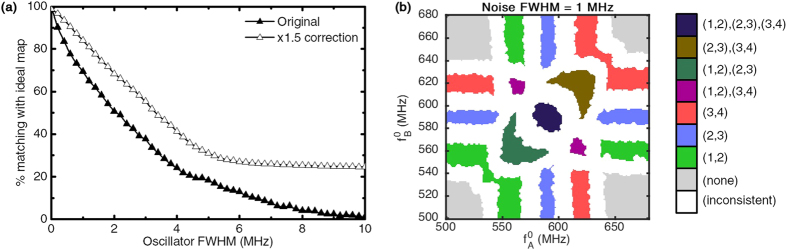
(**a**) Percentage of matching points to the ideal map with respect to noise FWHM for the original(▲) and the adjusted(Δ) systems. (**b**) An example of synchronization readout map under oscillator noise FWHM = 1 MHz in the original system.

The typical effect of noise is visible on the map of Fig. 3(b) obtained in the case of FWHM = 1 MHz: it erodes the surface of output synchronization pattern regions. Noise particularly affects the points where synchronization was weak, situated at the boundaries of the regions identified on reference map of Fig. 1(b). Figure 3(a) shows the percentage of matching points to the noiseless ideal map of Fig. 1(b) at different levels of noise. These results show that this architecture is resilient to relatively high noise levels, demonstrating 70% matching at FWHM = 1 MHz, corresponding to oscillators with *f*/FWHM ≥ 500. As a comparison, typical auto-oscillating magnetic nano-devices have shown *f*/FWHM ≥ 6000^34^ (FWHM lower than 100 kHz at 457 MHz), and mechanical oscillators^29^ can achieve *f*/FWHM ≈ 10^3^, which makes these technologies good candidates for this architecture.

In order to fully mitigate the effects of noise, we found that the distance between the natural frequencies of core oscillators can be increased. Figure 3(a) shows results for a case in which the couplings, distances between natural frequencies, and input frequency sweep ranges have been multiplied by a factor of 1.5(Δ). This system is notably more robust to noise than the initial system. However, this is a trade-off as it requires accessing a larger range of natural frequencies for the core oscillators, and ensuring stronger couplings. As a conclusion, the level of phase noise in oscillators should define the minimal interdistance between core oscillator natural frequencies.

### Effects of natural frequencies variability

Tuning the sizes and positions of the readout synchronization pattern regions requires the ability to set the natural frequency of every core oscillator. This is done either at design time by geometrical or material engineering of each oscillator, or by relying on the knowledge of the natural frequency tuning function *f*^ 0^(bias) of every oscillator. Both approaches are prone to device variability, which can lead to random shifts in the natural frequencies of the oscillators. As the distances between frequencies in the core network are critical parameters, this variability can induce behavioral changes.

**Figure 4:**
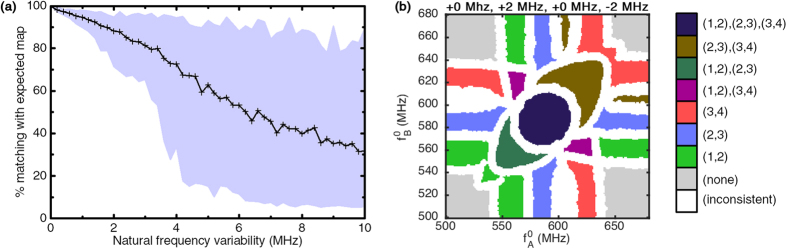
(**a**) Average matching with the expected map for 100 draws on different natural frequency uniform variability ranges, filled between best and worst cases encountered. (**b**) An example readout map where 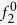 was shifted by +2 MHz and 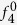 by −2 MHz: 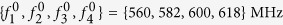.

To study the effects of natural frequency variability, the readout map was computed for increasing variability factors and compared to the reference map of Fig. 1(b). For increasing Δ*f*^0 ^ values, 100 possible outcomes are computed with core oscillator natural frequencies uniformly drawn in the range 
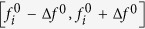
 where 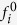 is the target natural frequency of oscillator *i*. Figure 4(a) shows the degree of matching with the expected readout map as a function of Δ*f*^ 0^. The solid curve represents the average matching for the 100 simulations, while the blue filled region shows the span between the worst and best matching values reached.

For low variability values, the worst case matching first slowly decreases up to approximately ±3 MHz, before dramatically falling below 50% for higher variability values. This worst case corresponds to a situation where the natural frequencies of a pair of core oscillators are brought close enough so that undesired synchronization appears even without input stimuli. Nevertheless, the worst case matching stays over 70% up to a variability of ±2 MHz, which corresponds to 10% of the initial difference between two consecutive core network natural frequencies.

An example of obtained readout map is shown on Fig. 4(b) in a case of 2 MHz variability. In this map, 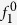 and 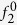 are slightly further apart than expected, which reduces the region 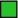 in which they can synchronize. On the contrary, 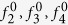 are closer than expected and regions 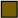 where all three are synchronized appear on the sides of the map.

These results show that the system is robust to moderate natural frequency variability, but that high variability on natural frequencies rapidly induces uncertainty on the system response. Again, we conclude that knowledge of the variability level of the system defines the minimal natural frequency difference between consecutive core oscillators.

In the presence of high variations, an effective approach could be to take inspiration from the reinforcement learning algorithms from the machine-learning expertise, and use the readout to implement a feedback of the oscillator biases in order to achieve the correction of the natural frequency values^8^,^52^, taking advantage from the tunability of the core oscillators.

### Effects of coupling constants variability

Variability on the coupling values can arise from variability in inter-oscillator distances (in case of proximity coupling effects), variability in electrical connections, variability in the signal amplitude they emit or variability in their individual response to stimuli^40^,^45^.

The consequences of such variability were studied by computing the readout map for increasing variability amplitudes in the coupling strengths. For each value of variability amplitude *μ* ranging between 0 and 100%, 100 simulations were run after randomly drawing individual couplings *k*_*a,b*_ in the uniform range 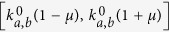 where 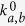 is the initial coupling without variability.

**Figure 5:**
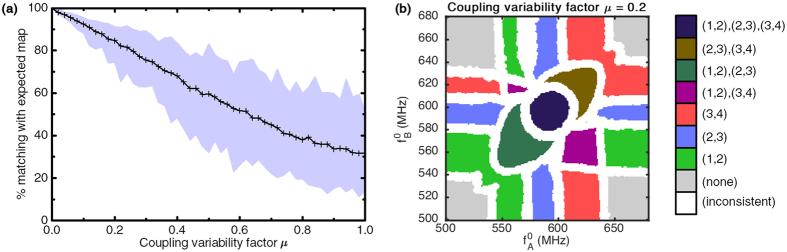
(**a**) Average matching with the expected map for 100 draws with different coupling uniform variability amplitudes *μ*, filled between best and worst cases encountered. (**b**) An example readout map for *μ* = 0.2.

A typical map obtained under a coupling variability *μ* = 20% is shown in Fig. 5(b). It shows that coupling variability has an influence on the size and shape of the synchronization regions in the readout map. Notably, it has a significant impact on regions corresponding to the synchronization of a single pair of oscillators. Indeed, the smaller the coupling between the two oscillators, the smaller the input frequency range in which they will synchronize is. The readout map also shows that the core network no longer responds symmetrically to the two input stimuli, due to variability in input-core couplings.

The average matching with the expected readout as a function of the coupling strength variability amplitude is shown in Fig. 5(a). The filled region represents the span between the worst and best matching rates encountered. Results show that the network is robust to coupling variability. Indeed, even 100% variability does not fully hamper the function of the recognition process, as no sudden breakdown is observed. A reasonable 20% variability, even in the worst case scenario that was simulated, leads to more than 70% matching with the expected readout map. This robustness can be attributed to the existence of redundant couplings in the core network, that tends to even out local coupling variations. These results are very positive in the context of nanotechnologies, for which precise engineering of individual couplings in a network are complex to achieve.

### Effects of coupling phase shifts

Coupling between oscillators can arise from many different phenomena: magnetic interaction^35^, electrical coupling^30^,^37^, mechanical coupling^31^. Notably, couplings can have both a conservative and a dissipative component^1^,^5^, and sometimes involve delays. To fully account for the different types of interactions, a non-zero uniform coupling phase shift term *ϕ*_*i,j*_ = *ϕ* is added in the solved Kuramoto equation 1, and its influence on the system is assessed through the following simulations. The readout map was simulated for different values of the coupling phase-shift term *ϕ*, in the ideal case of noiseless oscillators and with no variability.

**Figure 6:**
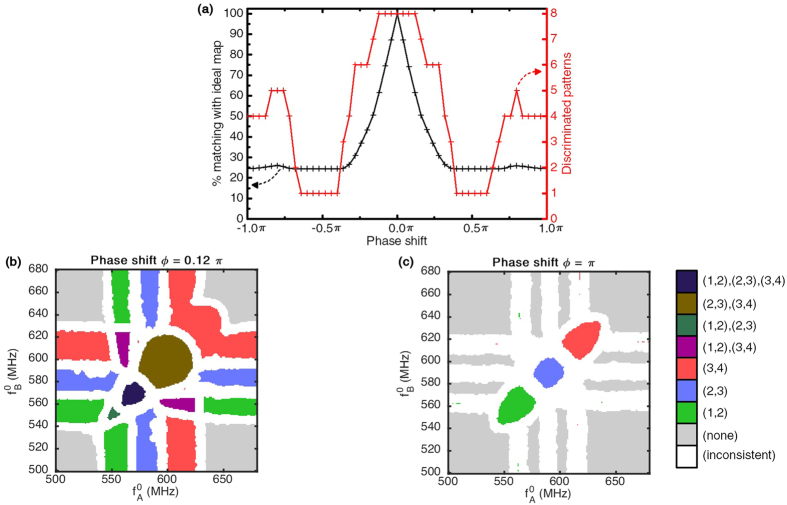
(**a**) Matching with the ideal map(+) and total number of different discriminated patterns(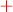) in the readout map as a function of phase shift *ϕ*. (**b**) An example readout map for a phase shift *ϕ* = 0.12*π*. (**c**) Example readout map for *ϕ* = *π*.

Figure 6(a) shows the evolution of the degree of matching of the readout map to the ideal map of Fig. 1(b), as well as of the number of different discriminated synchronization patterns, as a function of the coupling phase shift value. Results show that the phase-shift has significant effects on the response of the system, as the matching with the reference map drops with increasing *ϕ*. Notably, it drops under 50% for |*ϕ*| > *π*/6. It then reaches a plateau at 25% of matching for |*ϕ*| > *π*/3 where only the areas of the map where no synchronizations are present are consistent with the ideal readout map.

The number of discriminated synchronization patterns is also strongly affected by the appearance of a coupling phase-shift. The maximum number, 8, holds for only small phase-shifts, and then progressively falls as |*ϕ*| increases toward 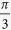, for which all the synchronizations break. As shown by the map of Fig. 6(c), when |*ϕ*| approaches *π*, we observe that only synchronization of pairs of oscillators arises, in the regions for which the two inputs have close frequencies.

The map obtained for *ϕ* = 0.12*π* is shown in Fig. 6(b). We can see that for this phase-shift, the synchronization pattern region shapes are already significantly deformed with regards to Fig. 1(b), especially when several pairs of core oscillators are involved. Indeed, while interaction phase-shifts do not prevent synchronization between two oscillators, frustrations a rise when several oscillators are involved in the synchronization process as seen in other contexts in refs 53 and 54. This phenomenon is critical in the context of the stimuli-induced core oscillator synchronizations, as at least three oscillators (2 core and 1 input) are involved.

As a conclusion, as the engineering of the pattern recognition architecture using a network of oscillators relies strongly on the ability to synchronize more than two oscillators, the phase relation between synchronized oscillators should be carefully cared about. The use of delay lines or reactive components in the network can in particular be integrated in the design process to solve this issue by bringing the coupling phase shift back to zero for optimal performance. Technological solutions allowing this can be found in refs 37, 53, 55 and 56.

### Effects of oscillator non-linearity

Non-linear behavior, *i.e.* phase-amplitude coupling, is a common property of nano-oscillators. It manifests itself through a frequency dependence on the oscillation amplitude. This non-linearity has a strong influence on the synchronization efficiency as it amplifies the effects of interactions^42^. In the following, we assess the consequences of non-linearity on the behavior of our pattern recognition architecture.

We now model the oscillators of the system by the following set of coupled equations:


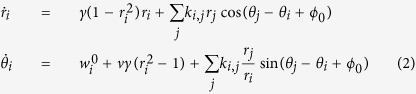


where *r*_*i*_ is the amplitude of oscillator *i, γ* is the damping coefficient for radius deviation, *v* is the dimensionless nonlinear frequency shift that quantifies the non-linearity, and *ϕ*_0_ is the coupling phase that depends on the physical nature of the coupling mechanism. This is a conventional way to model nonlinear oscillators, as described in ref. 42. The phase equation of this model reduces to the Kuramoto equation 1 in the absence of nonlinearity (*v* = 0). When phase-amplitude coupling is involved, it can be shown that a pair of oscillators interact with an increased effective coupling term: 
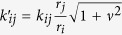
. The second effect of the nonlinearity implies a contribution to the effective coupling phase-shift, that then verifies:


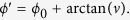


**Figure 7:**
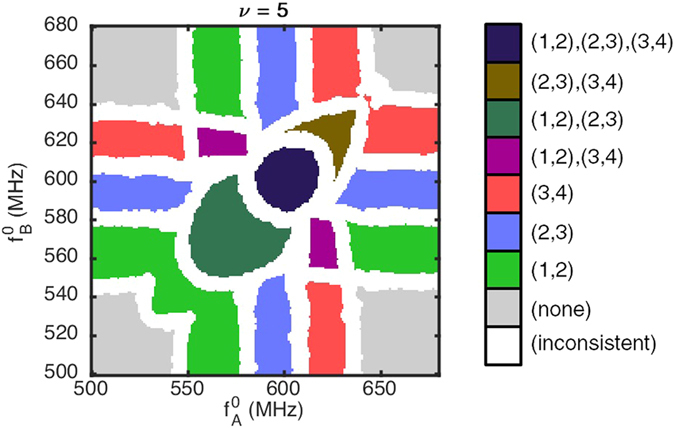
Readout map obtained for an oscillator network with conservative coupling (*ϕ*_0_ = −*π*/2), dimensionless non-linear frequency shift *v* = 5 and coupling strengths reduced by a factor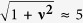 compared to the linear network.

The choice of a too strong coupling is detrimental for the recognition abilities of the network, this effective increase should be conveniently leveraged to allow the use of weaker physical couplings at design time, or allow a larger spacing between core oscillator natural frequencies. As an illustration, we show in Fig. 7 the readout map obtained in a case when the coupling between the oscillators is conservative (*ϕ*_0_ = −*π*/2) and with a strong non-linearity *v* = 5, for which all coupling strengths *k_ij_* were reduced by a factor 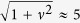. This rescaling allows to come back to effective coupling strengths close to those of Fig. 1(b). However, the map appears deformed as anticipated due to the non-zero effective phase-shift (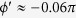 for *v* = 5).

We conclude that the nonlinearity effects are interestingly beneficial for the design of the introduced pattern recognition architecture. By artificially increasing the coupling strength, it allows the use of even more weakly coupled networks, but mostly it will allow to increase the spacing between core oscillators’ natural frequencies, that has strong interest to mitigate the effects of phase noise and variability.

In case of a conservative coupling, strong non-linearities can also be beneficial to push the effective coupling phase towards zero. In general cases, effects of the non-linear behavior on this phase-shift should be carefully considered as a function of the physical origin of the coupling, and the subsequent *ϕ*_0_ value, aiming for an effective phase-shift as close to 0 as possible.

### System scalability

We observed that our reference architecture composed of four core oscillators and two input oscillators already allows discriminating up to eight different classes of stimuli. We now study the evolution of the maximum number of synchronization patterns reached by the proposed architecture as it scales by increasing the number of both core and input oscillators.

During the readout phase, synchronization is evaluated between pairs of core oscillators with consecutive natural frequencies. For *N*_*c*_ core oscillators, each one of the *N*_*c*_ − 1 pairwise evaluations can return a positive or a negative answer. The theoretical upper bound on the number of discriminated patterns can then be derived as the number of possible values taken by a binary word of *N*_*c*_ − 1 bits, i.e. 2^*Nc*−1^.

**Figure 8:**
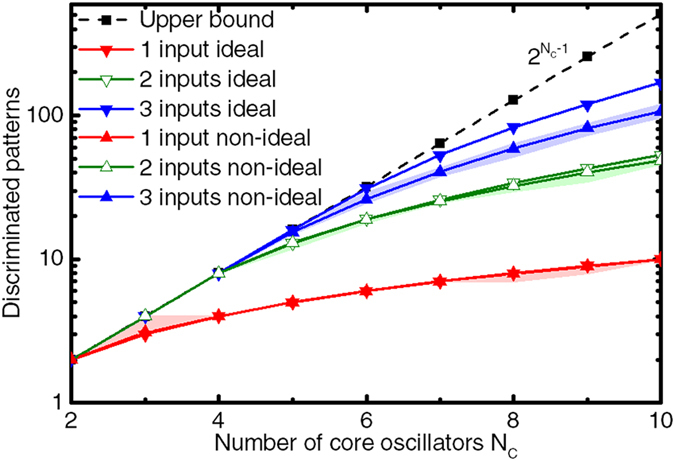
Number of different discriminated patterns in the readout map as a function of the number of core oscillators *N*_*c*_, for *N*_*i*_ = {1, 2, 3} input oscillators, as evaluated in an ideal case and in an average case with high noise and variability. The filled regions represent the span between the best and worst cases encountered during 10 random variability trials. The theoretical upper bound(■) corresponds to 2^*Nc*−1^.

Nevertheless, depending on the number of input oscillators, different proportions of these synchronization outcomes can be reached. To investigate the actual capacity of the system, we have computed the readout maps of the proposed circuit on GPU for different numbers of core oscillators and different numbers of input oscillators, and have counted the number of different unique available synchronization patterns in each of them. To keep the parameters of the system unchanged, we maintain a constant 20 MHz difference between consecutive natural frequencies in the core network. Note that an increase in the number of core oscillators is bounded by the availability of a larger range of natural frequencies. The number of dimensions of the computed maps is equal to the number of inputs. The ideal cases (without noise nor variability) in Fig. 8 show the number of discriminated patterns for the readout maps obtained for different numbers of ideal core and input oscillators, as well as the aforementioned theoretical upper bound.

These results show that the response of the ideal system is very rich as it displays an important number of different synchronization patterns. The number of observed synchronization patterns substantially increases both with the number of core oscillators, and with the number of input oscillators. More specifically, the number of patterns versus number of core oscillators follows the exponential theoretical upper bound curve before its increase starts slowing down. The maximum number of patterns stays equal to the theoretical upper bound longer when more inputs are present. This is due to the fact that a bigger number of groups of oscillators can be synchronized independently when more inputs are available, which increases the total number of patterns reachable by the system.

As we have seen that the presence of noise and variability in such oscillator-based computing architectures has an influence on the network’s synchronization behavior, it is important to assess the scalability of the system in a non-ideal case. We reproduced simulations including an important level of noise and variability: noise FWHM = 1 MHz, ±1 MHz variability on natural frequencies and 10% variability on the couplings, as defined in the previous sections. The non-ideal cases in Fig. 8 show the average number of discriminated synchronization patterns in the response maps obtained on 10 random trials in these conditions. The filled areas correspond to the span between the best and worst cases encountered during the random trials.

We observe that this level of noise and variability does not or barely affect the number of discriminated patterns in the 1 and 2 input cases, as well as in the 3 inputs case until *N*_*c*_ = 5. For higher *N*_*c*_ values, in the three-input case, the number of output synchronization patterns is reduced with regards to the ideal case: in the worst case *N*_*c*_ = 10, the number of stable patterns discriminated drops from 169 in the ideal case, to 107 on average. This loss is mainly due to the noise effects: the tested variability values, although high, have little effect on the number of synchronization patterns. The patterns suppressed by noise tend to be the smallest, therefore less-reliable, patterns.

Nevertheless, we see in Fig. 8 that the presence of noise and variability does not fundamentally affect the scaling capability of the system, but requires avoiding using unreliable patterns of the response map. We should also remark that, in the presence of noise and variability, the previously mentioned frequency spacing criteria also limit the scalability of the system provided that the accessible natural frequency range for core and input oscillators is limited.

Overall, these results show that the maximum number of synchronization patterns of the proposed system is high and scales well with the number of oscillators, which makes the system attractive for many-class classification and associative memories.

### Effects of geometrical constraints in the network

Uniform all-to-all coupling is a straightforward hypothesis when considering networks of oscillators. Nevertheless, densely packed networks of oscillators also offer the possibility to leverage coupling through proximity effects. In such a situation, the distance between oscillators can affect their coupling strength and/or induce phase shifts in their signals. The geometrical arrangement of devices then appears as a fundamental consideration. It is then interesting, in the nano-device context, to assess the robustness of the studied computing scheme in the case of non-uniform couplings.

#### Spatially decaying coupling strengths

Proximity couplings usually involve short characteristic interaction distances. Coupling strength then decreases with the physical distance between two oscillators. This applies for example to mechanical couplings through acoustic waves^31^, to spin wave coupling of spintronic oscillators^36^,^57^, to optical couplings^28^ or to couplings through dipolar electrical or magnetic fields^35^.

To assess this effect, we simulate our 2-input architecture considering 10 core oscillators arranged along a line, sorted by increasing natural frequency, and with core-core coupling strengths 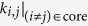 decreasing exponentially with distance:


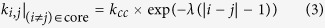


where *λ* is the spatial coupling decay factor. For generality, input-core couplings are left unchanged.

**Figure 9:**
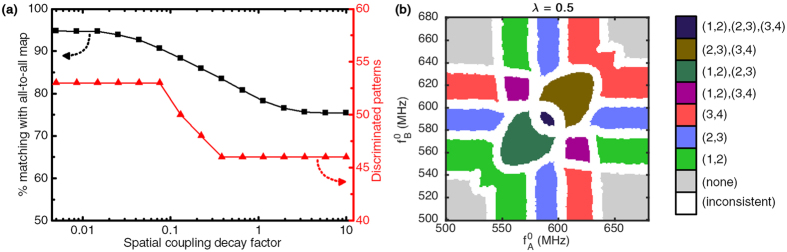
(**a**) Matching with the response map in the uniform all-to-all coupling case(■), and number of discriminated patterns(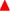) in the resulting map for *N*_*c*_ = 10 core oscillators as a function of the spatial coupling exponential decay factor. The oscillators are assumed to be arranged along a line, spaced by one distance unit, and ordered by increasing natural frequency. (**b**) Example response map obtained for *N*_*c*_ = 4 core oscillators, *N*_*i*_ = 2 input oscillators and *λ* = 0.5.

Figure 9(a) shows the number of discriminated patterns in the response map, as well as its matching to the map with all-to-all uniform couplings (*λ* = 0), as functions of the spatial coupling decay factor *λ*. The graph shows that the system behavior changes minimally up to *λ* = 0.075, where it shows 90.6% matching to the all-to-all map with all its 53 patterns still present. The number of discriminated patterns then drops and stabilizes to 46 for *λ* > 0.4 and the matching with the all-to-all map drops and stabilizes to 75.4% for *λ* > 5.8.

In order to illustrate the effects of such a decay, we also compute an example *N*_*c*_ = 4 core oscillator response map under a high decay factor (*λ* = 0.5). The corresponding response map Fig. 9(b) shows that only the 4-oscillator pattern 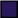 disappears as the coupling decay increases. On the other hand, patterns involving a single input and 2 core oscillators, 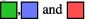 remain unaffected.

Patterns involving at most single pairs of synchronized oscillators remain unaffected because they rely on first-neighbor couplings only. On the other hand, patterns involving at least one group of more than 2 synchronized oscillators are affected because long range interactions between distant neighbors in the group contribute to their stability.

These results show that in a configuration where couplings are decaying in space, or even limited to first neighbors, the system only loses a small portion of its capacity, as most of the synchronization patterns can be stabilized by short-range interactions alone.

#### Spatially-increasing coupling phase shifts

When coupling in the network involves propagating waves, the distance between two oscillators can also induce non-negligible time delays, and therefore distance-dependent coupling phase shifts. They can also be induced by material non-linearities^58^.

To account for distance-dependent coupling phase shifts we simulate the 2-input architecture with 10 core oscillators arranged spatially along a line, ordered by increasing natural frequency, and with a distance-dependent coupling phase shift term between core oscillators 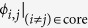:


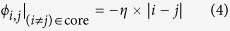


where *η* represents the phase shift per unit distance.

**Figure 10:**
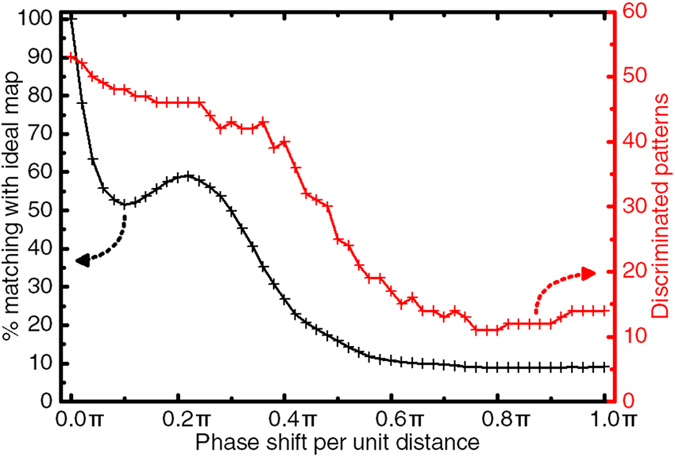
Matching with the ideal map(+) and total number of different discriminated patterns(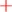) in the readout map as a function of the phase shift per distance unit, for *N*_*c*_ = 10 core oscillators. Oscillators are assumed to be arranged along a line, spaced by one distance unit, and ordered by increasing natural frequency.

Figure 10 shows the number of discriminated patterns in the response map, as well as its matching to the ideal *η* = 0 map, as functions of the phase shift per unit distance *η*. Similarly to the uniform phase shift case, the response map of the system is heavily altered by distance-related phase shifts. For *η* = 0.04*π*, the matching with the ideal map already drops to 63.4%, as the shape of pattern regions is changing, but only 3 patterns are not observed anymore. Generally, while the matching to the ideal *η* = 0 map quickly decreases below 60%, the number of stable patterns stays high, over 40, for moderate values of *η* (<0.4*π*). A locally optimal situation is observed around *η* = 0.22*π*, which is related to system symmetries. Patterns involving only 2 core oscillators synchronized with a single input are again the most resilient to these phase shifts, while the apparition of frustrations quickly destabilizes patterns involving multiple synchronized oscillators.

Overall, to keep the maximal capacity of the system, accumulated contributions to the phase-shifts between every pair of oscillators should be brought as close to zero as possible. Considering propagation-related effects, a careful distribution of oscillators in space should be considered to ensure target phase-shift. It is however expected that, in case of decaying coupling, the influence of distance-dependent phase-shift should be lower.

## Discussion

In this work, we have shown that a network of nano-oscillators can be used to achieve recognition/classification operations by relying on the rich synchronization dynamics of its oscillators.

In an ideal situation with noiseless oscillators and in absence of variability issues, it allows the classification of stimuli in a large number of classes. Even if its capacity is diminished as compared to an ideal case, this computing architecture is also fully compatible with noise levels and device variability corresponding to current achievements in nanotechnology. Resilience to noise and device variability is a widespread feature of neural network-inspired architectures^59^, and a strong advocate for their use in conjunction with nanotechnologies. In the case of our system, the relaxed nature of synchronization evaluation further helps the system deal with noisy situations.

A challenge for the design of oscillator-based computing units and their scalability is the minimal number of oscillators that is required to achieve non-trivial computational tasks. In this work, we have shown that this architecture can allow for complex classification tasks even with a network of a reduced number of oscillators. It is able to discriminate oscillating stimuli into a number of classes that scales rapidly with the number of core oscillators, even in situations with high phase noise and variability. With this fast increase of the computational complexity with the number of oscillators, complex cognitive tasks can already be achieved with a limited number of oscillators, as illustrated in this study in the case *N*_*c*_ ≤ 10 oscillators.

Concerning scalability, our study has highlighted network design rules, for which the minimal natural frequency spacing among core oscillators has to be set in agreement with the expected noise and variability amplitudes. As a consequence, the accessible natural frequency range appears as a crucial parameter during the architecture design.

Nanodevice-based oscillators are generally non-linear^42^,^43^. We have shown that these non-linearities – i.e. phase-amplitude coupling – allow the use of weaker couplings in the core computing network, by increasing the oscillators’ synchronization capabilities. They allow the spacing between core oscillators frequencies to be increased, but also contribute to the phase-shifts between synchronized pairs of oscillators.

Such phase-shifts, that also arise from the global properties of the coupling, appear to be the most important practical challenge towards achieving oscillator network computation. They are responsible for the appearance of frustrations that restrict synchronization of more than two oscillators, and reduce the global synchronization capabilities of the network. Therefore, this point should receive special attention for the choice of ideal technology as well as ideal coupling type and geometry in the design of the network. Coupling phase shifts can also be adjusted at design time: for instance, in the case of high frequency oscillators, proper transmission line design can introduce phase delays between oscillators so that phase shifts are ideally brought back to zero^37^,^53^,^55^,^56^.

The computing scheme described through this work was also shown to be compatible with different oscillator coupling strategies. While all-to-all coupling guarantees the highest classification capacity, coupling through proximity effects, prone to appear in nano-device networks, can also be leveraged with high computing capacity.

Another challenge for scalability in the context of nanotechnology implementation is the number of detection circuits. In the proposed architecture, the number of necessary detection circuits is limited to *N*_*c*_ − 1, scaling linearly with the number of oscillators.

A proposal to achieve advanced classification could also be to rely on the juxtaposition of several small core networks, trained independently to discriminate complementary subsets of patterns. From a nanotechnology point of view, this also avoids the complex fabrication of large networks of nano-objects and would facilitate the training of the networks.

This work contributes to a recent vision of nanodevice-based computing where nanodevices would be “more than a switch”^60^. We can exploit the whole intrinsic physics of the devices for achieving significant computing tasks with a minimal number of devices. The most important challenge for the success of this vision will be to demonstrate practically scaling and programming capabilities.

## Methods

Simulations not involving noise use 4th order Runge Kutta integration. When noise is involved, the Euler-Maruyama SDE integration scheme is used. To ensure convergence, decreasing time-steps were tried until no change on the phase evolution of individual oscillators was observable, and the limit time-step was further divided by 10. The final time-step used that ensures convergence of both integration schemes is dt = 100 ps. The numerical scheme used for the Kuramoto equation is given in equation 5, where 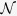 is the Gaussian distribution.


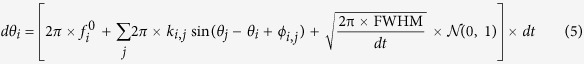


The total simulation time was 1 μs of which the first 0.5 μs are the transient stabilization time after which synchronization detection counting starts. This waiting time was chosen to ensure the stabilization of the dynamics on 1,000 simulation runs of the reference system with different random initial conditions. The total simulation time corresponds to approximately 600 periods of the oscillators and is chosen to be a realistic scale for real-life implementations. Two oscillators are considered synchronized when the final absolute value of their counter is strictly less than 6, that is less than about 2% difference in their number of periods^51^.

The computed 4 core oscillator maps are 200 × 200 points with 10 simulations per point, which amounts for 400,000 simulations. To keep the same resolution, the maps of the scaling study and 10 core oscillator maps were computed using 333 × 333 × 10 = 1,108,890 simulations. Since all simulations are independent, they were run in parallel on nVidia Tesla K40m GPUs. The simulation code is written in C++ and uses the CUDA Thrust library.

## Additional Information

**How to cite this article**: Vodenicarevic, D. *et al*. A Nanotechnology-Ready Computing Scheme based on a Weakly Coupled Oscillator Network. *Sci. Rep.* **7**, 44772; doi: 10.1038/srep44772 (2017).

**Publisher's note:** Springer Nature remains neutral with regard to jurisdictional claims in published maps and institutional affiliations.
